# Anticancer Effects of Mitoquinone via Cell Cycle Arrest and Apoptosis in Canine Mammary Gland Tumor Cells

**DOI:** 10.3390/ijms25094923

**Published:** 2024-04-30

**Authors:** Ran Lee, Won-Young Lee, Hyun-Jung Park

**Affiliations:** 1 Department of Livestock, Korea National University of Agriculture and Fisheries, Jeonju-si 54874, Republic of Korea; ranran2424@gmail.com (R.L.); leewy81@korea.kr (W.-Y.L.); 2 Department of Animal Biotechnology, College of Life Science, Sangji University, Wonju-si 26339, Republic of Korea; 3 Department Smart Life Science, College of Life Science, Sangji University, Wonju-si 26339, Republic of Korea

**Keywords:** antioxidants, cancer, canine, mammary gland, apoptosis

## Abstract

Treating female canine mammary gland tumors is crucial owing to their propensity for rapid progression and metastasis, significantly impacting the overall health and well-being of dogs. Mitoquinone (MitoQ), an antioxidant, has shown promise in inhibiting the migration, invasion, and clonogenicity of human breast cancer cells. Thus, we investigated MitoQ’s potential anticancer properties against canine mammary gland tumor cells, CMT-U27 and CF41.Mg. MitoQ markedly suppressed the proliferation and migration of both CMT-U27 and CF41.Mg cells and induced apoptotic cell death in a dose-dependent manner. Furthermore, treatment with MitoQ led to increased levels of pro-apoptotic proteins, including cleaved-caspase3, BAX, and phospho-p53. Cell cycle analysis revealed that MitoQ hindered cell progression in the G1 and S phases in CMT-U27 and CF41.Mg cells. These findings were supported using western blot analysis, demonstrating elevated levels of cleaved caspase-3, a hallmark of apoptosis, and decreased expression of cyclin-dependent kinase (CDK) 2 and cyclin D4, pivotal regulators of the cell cycle. In conclusion, MitoQ exhibits in vitro antitumor effects by inducing apoptosis and arresting the cell cycle in canine mammary gland tumors, suggesting its potential as a preventive or therapeutic agent against canine mammary cancer.

## 1. Introduction

Canine mammary gland tumors (CMT) represent the most prevalent tumors in female canines, and the treatment of these tumors is pivotal for the welfare and well-being of affected animals. Dogs have evolved from mere pets to cherished family members, underscoring the significance of addressing their health concerns. In European studies, CMT accounts for 0.25% of female dogs in Italy (1997–1998) [1], 0.21% of female dogs in the population of insured dogs in the UK (1997–1998), and 1.11% female dogs in Sweden (1995–2002) [1,2,3]. Previous reports indicate a comparable distribution of benign (53%) and malignant (47%) tumors among female dogs [4], with shared clinical, genetic, and pathological characteristics alongside epidemiological, environmental, and biological factors [5,6]. Although adjuvant chemotherapies like doxorubicin [7,8], carboplatin [9,10], mitoxantrone, and paclitaxel have been explored for malignant neoplasms, large-scale studies reveal limited clinical efficacy, and agents such as gemcitabine [11,12,13], doxorubicin, docetaxel [14], and mitoxantrone [15] are associated with high recurrence rates and unfavorable prognoses in CMT patients.

Traditional cancer treatment protocols involving surgery, chemotherapy, and other interventions can prolong the lives of dogs diagnosed with mammary gland cancer. Thus, this study delves into exploring the anticancer effects of MitoQ, either as an adjuvant treatment or a standalone anticancer agent, for addressing mammary epithelial cell tumors in dogs [3,7].

The excessive generation of oxidative stress correlates with cancer growth [16], with the rise in reactive oxygen species (ROSs) in cancer cells being metabolism dependent. Dysregulation of oncogene signaling pathways, ROSs, and antioxidants influences anticancer therapy outcomes [17]. Mitochondria, the sites of respiration and oxidative metabolism, play a critical role in ROS generation, rendering mitochondria-targeting agents promising candidates for anticancer therapy with minimal toxicity [18]. Cancer cells can adapt to elevated ROS levels [19], surviving DNA damage, protein toxicity, and metabolic stress [20], highlighting the significance of antioxidants in anticancer therapy [21]. ROSs impact signal transduction and signaling pathway regulation, leading to tumorigenesis, aberrant proliferation, and cell metastasis and migration [22]. ROS-mediated DNA oxidation activates tumorigenesis and deactivates tumor suppressor genes [23], with excessive tumorigenesis resulting from the stimulation of the PI3K/AKT and MAPK signaling pathways alongside ROS-mediated inhibition of protein phosphatases [24,25,26]. In tumors, some malignant cells resort to glycolysis for energy production due to oxygen availability fluctuations and changes in cellular properties [27]. Mitochondria maintain tumor oxidative capacity when such mutations occur, terminating glycolysis [28,29].

Tumor metastasis poses a significant and often fatal challenge to cancer treatment [30], with studies indicating the release of approximately 1 × 10^6^ tumor cells per gram of tumor mass into circulation daily [31]. These metastatic progenitor cells induce tumor metastasis in various organs of mice, with mitochondria associated with electron transport chain (ETC) superoxide playing a crucial role in promoting tumor cell migration, invasion, clonogenicity, and metastasis. Mitochondrial superoxide elimination is pivotal for prevention [32,33].

MitoQ, a mitochondria-targeted antioxidant, has been investigated for its potential anticancer effects in humans. Mitochondria, essential organelles responsible for energy production, also regulate cell death and survival [34,35]. MitoQ, comprising coenzyme Q10 and the lipophilic triphenylphosphonium cation (TPP+), exhibits superior antioxidant activity compared to non-target antioxidants [36]. MitoQ’s ubiquinone molecules shield mitochondrial membranes from lipid peroxidation [37], being introduced into the lipid bilayer of the matrix and reduced to ubiquinol from complex II [38]. MitoQ participates in the antioxidant signaling pathway, converting generated superoxide into water, and being recycled without destruction, efficiently reducing mtO2-signals [37]. Notably, MitoQ concentrates up to 100-fold on the matrix surface of the mitochondrial inner membrane [27] and exists in ubiquinone and ubiquinol forms [39]. MitoQ scavenges superoxides from the ETC and acts as an antioxidant, converting peroxides to water. Unlike endogenous coenzyme Q10, MitoQ evades oxidation by ETC complex III, undergoing recycling rather than destruction after the process, preserving mitochondrial function [40,41]. This unique property enables MitoQ to efficiently reduce mtO2- signals and maintain mitochondrial function [41].

In contrast to coenzyme Q10, MitoQ remains unoxidized by ETC complex III, making it effective in normoxic and hypoxic tumors [37]. MitoQ’s intramitochondrial storage and activity stem from continuous recycling of ubiquinol moieties from the respiratory chain into active antioxidants [41], capable of repairing or mitigating cellular damage inflicted by oxidative stress during in vitro culture [42,43,44]. MitoQ’s antioxidant properties have spurred extensive research in cancer [34], muscular atrophy [45], cardiovascular diseases [46], and neurodegeneration [47]. In dogs, changes in estrogen receptors primarily contribute to mammary cancer, alleviated via female spaying. However, mammary cancer remains prevalent in intact female dogs [48].

This study explores MitoQ’s impact on cancer cell death, migration, and signaling pathways in canine mammary tumor cells, specifically the CMT-U27 and CF41.Mg cell lines characterized as mammary cancer cells [49,50]. Our findings suggest MitoQ’s potential as a safe and effective treatment for canine mammary tumors, significantly reducing tumor cell viability and migration capabilities while modulating various cellular signaling mechanisms.

## 2. Results

### 2.1. Effects of MitoQ on Cell Viability and Proliferation in Canine Mammary Tumor Cells

**Figure 1:**
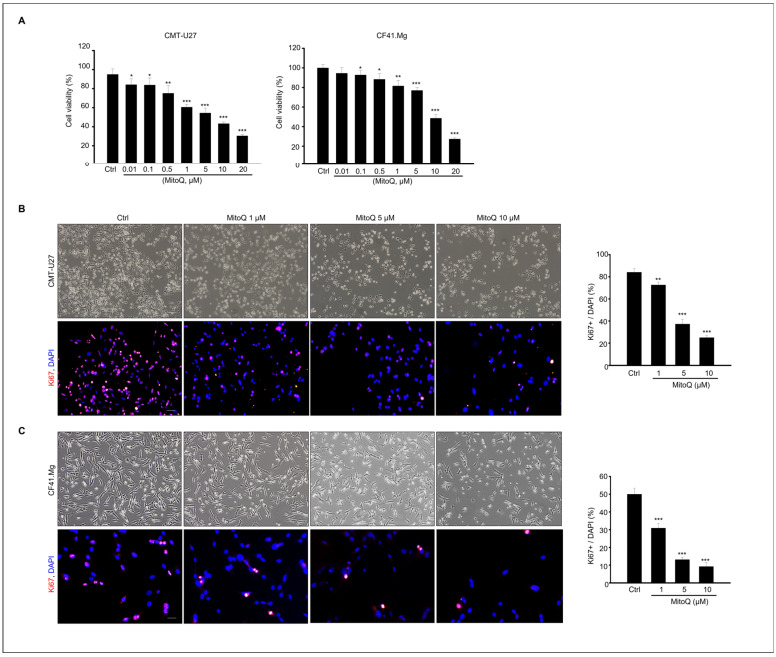
MitoQ inhibits viability of canine mammary gland tumor cells, CMT-U27, and CF41 Mg. cells. (**A**) Cell viability was assessed in CMT-U27 and CF41. Mg cells following treatment with MitoQ (0–20 μM) or DMSO (vehicle) as a control after 24 h of culture. The graph presents the mean ± SEM of three independent experiments. Microscopic images of Ki67-positive cells in (**B**) CMT-U27 and (**C**) CF41. Mg cells were obtained after 24 h of exposure to different concentrations of MitoQ (0, 1, 5, and 10 µM). The relative percentage of Ki67-positive cells is depicted on the graph. The data represent the mean ± SEM of three different experiments. * *p* < 0.05; ** *p* < 0.01; *** *p* < 0.001, compared to controls. Scale bar = 50 µm.

Initially, to evaluate the cytotoxic potential of MitoQ, we examined the viability of CF41.Mg and CMT-U27 cells using a cell viability assay. As depicted in Figure 1A, MitoQ concentrations of 1, 5, 10, and 20 µM hindered CMT-U27 cell viability significantly, by 75%, 60%, 54%, 43%, and 30%, respectively. Similarly, assessment of MitoQ’s cytotoxic impact on CF41.Mg cells demonstrated significant viability reduction, with concentrations of 1, 5, 10, and 20 µM leading to viability decreases of 81%, 76%, 48%, and 27%, respectively (Figure 1A). Furthermore, we investigated the effects of MitoQ on the proliferation of CMT-U27 and CF41.Mg cells. Based on the initial viability results (Figure 1A), MitoQ treatment concentrations ranging from 0 to 10 µM were selected for subsequent experiments. Utilizing Ki67 staining to confirm cell proliferation, immunofluorescence imaging revealed a notable reduction in proliferation in both cancer cell cultures following 24 h of MitoQ treatment. The graph illustrates the percentage of Ki67/DAPI in MitoQ-treated cells, demonstrating a dose-dependent decrease in Ki67-positive cells in both cell types after MitoQ treatment. Particularly, concentrations of 5–10 µM of MitoQ led to a reduction in cell proliferation of 40% or less compared to untreated cells in both cell types (Figure 1B,C).

### 2.2. Apoptotic Effects of MitoQ on Canine Mammary Gland Tumor Cell Culture

**Figure 2:**
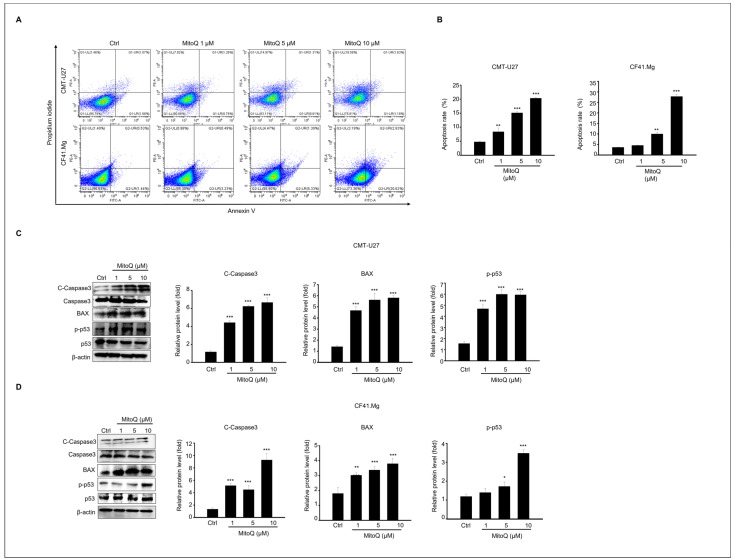
Apoptotic response to MitoQ in canine mammary gland tumor cells. (**A**) Apoptosis in CMT-U27 and CF41. Mg cells was detected after 24 h of treatment with 0, 1, 5, and 10 µM of MitoQ using Annexin V-FITC/PI staining and analyzed using flow cytometry. (**B**) The graph illustrates the apoptosis rate of CMT-U27 and CF41. Mg cells as mean ± SEM. Immunoblot results of cleaved caspase-3, caspase-3, BAX, pp53, p53, and β-actin from (**C**) CMT-U27 and (**D**) CF41. Mg cell lysates. The graph represents the relative protein expression levels normalized to the inactive form or β-actin as mean ± SEM. N = 4, * *p* < 0.05, ** *p* < 0.01; *** *p* < 0.001.

Flow cytometry analysis using annexin-V and PI staining was conducted to assess cell death induced by MitoQ treatment. As illustrated in Figure 2B, the proportion of apoptotic cells increased 24 h after MitoQ treatment in CMT-U27 and CF41.Mg cultures. MitoQ concentrations of 1–10 μM elevated the apoptotic rate of CMT-U27 cells significantly, by 8.4%, 15.6%, and 20.3%, respectively. Similarly, the apoptosis rates of MitoQ (1–10 μM) in CF41.Mg cells were 4.6%, 10.3%, and 28.6%, respectively, in a concentration-dependent manner.

Additionally, compared to the control group, the expression levels of pro-apoptotic proteins, including cleaved caspase-3, BAX, and phosphorylated p53, were analyzed via immunoblotting. All these protein levels were significantly upregulated, reaching their peak at a 10 μM concentration of MitoQ in CMT-U27 and CF41.Mg cells (Figure 2C,D).

### 2.3. Effects of MitoQ on Cell Cycle Arrest and Expression of Cell Cycle Regulatory Proteins in Canine Mammary Gland Tumor Cells

**Figure 3:**
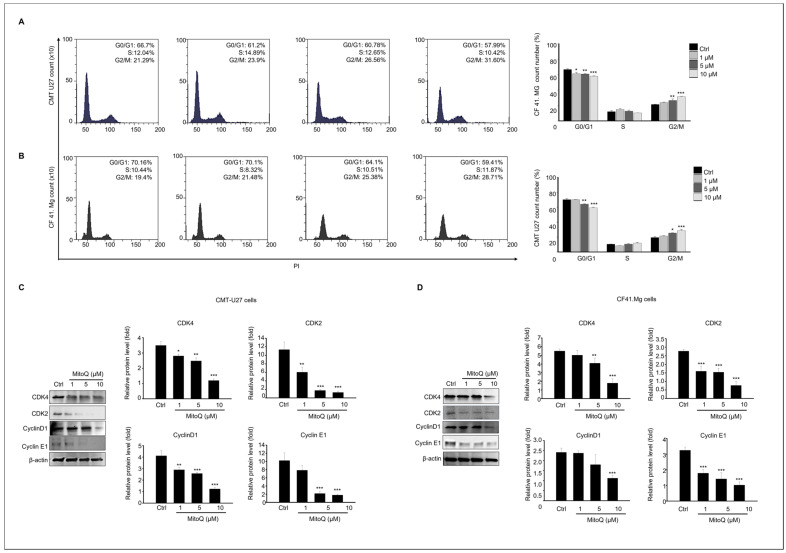
Cell cycle arrest induced by MitoQ in canine mammary gland tumor cell culture. The cell cycle profiles of (**A**) CMT-U27 and (**B**) CF41. Mg cells following exposure to MitoQ were analyzed using flow cytometry after PI staining, and the statistical analysis of cell cycle distribution in both cell lines treated with MitoQ is depicted in the graph. Protein expression levels of CDK4, CDK2, Cyclin D1, Cyclin E1, and β-actin from (**C**) CMT-U27 cells and (**D**) CF41. Mg cells are presented. The graph shows the relative protein expression levels as mean ± SEM. n = 4, * *p* < 0.05; ** *p* < 0.01; *** *p* < 0.001.

To assess the influence of MitoQ on the cell cycle and apoptosis profiles of canine mammary gland tumor cells, we analyzed the cell cycle distribution of CMT U27 and CF41.Mg cells after 24 h of MitoQ treatment using flow cytometry (Figure 3). The results demonstrated a significant dose-dependent decrease in the proportion of cells in the G0 and G1 phases in both CMT U27 and CF41.Mg cells compared to the control group following MitoQ treatment. Moreover, the proportion of cells in the G2/M phase significantly decreased in both cell types after 24 h of MitoQ treatment (Figure 3A,B). Subsequently, to understand the mechanism underlying MitoQ-induced cell cycle arrest in canine mammary tumor cells, we evaluated the expression levels of cell cycle regulatory proteins through immunoblotting. Compared to the control group, MitoQ treatment markedly reduced the protein levels of CDK4, CDK2, cyclin D1, and cyclin E1, key regulators of the G1/S transition, in both CMT U27 and CF41.Mg cells (Figure 3C,D).

### 2.4. Effects of MitoQ on Canine Mammary Cancer Cell Migration

**Figure 4:**
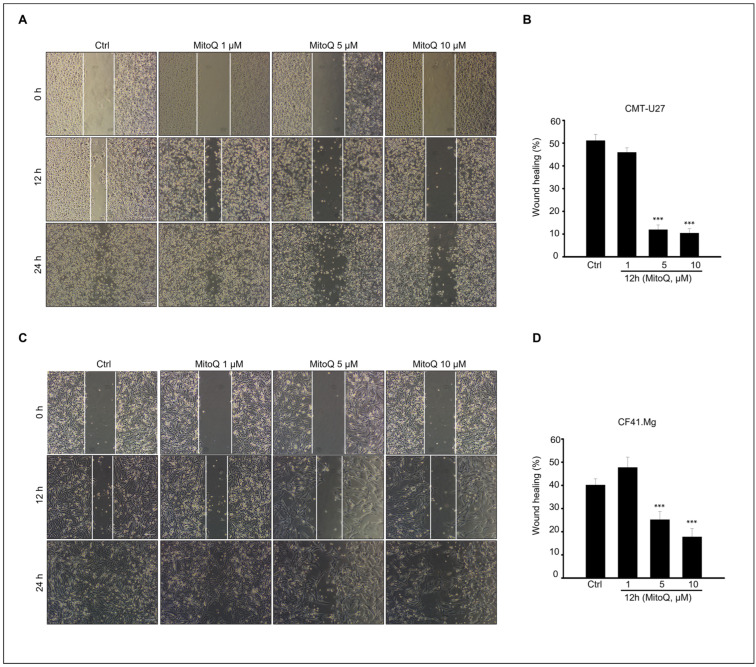
Inhibition of growth and migration of canine mammary gland tumor cells by MitoQ. (**A**) Microscopic images illustrating migration of CMT-U27 cells treated with 0, 1, 5, and 10 μM of MitoQ. Scale bar = 100 µm. (**B**) The graph assesses the wound-healing area of CMT-U27 cells. (**C**) Microscopic images demonstrating invasion of CF41. Mg cells treated with 0, 1, 5, and 10 μM of MitoQ. Scale bar = 100 µm. (**D**) The graph evaluates the wound-healing area of CF41. Mg cells. *** *p* < 0.001, scale bar = 100 µm.

To evaluate the inhibitory effect of MitoQ on the migration and invasion abilities of canine cancer cells, a scratch migration assay was conducted (Figure 4). The results demonstrated that MitoQ treatment significantly reduced the migration of CMT-U27 and CF41. Mg cells, as observed in the scratch wound assay (Figure 4A,C). The graph illustrates the percentage of wound healing (Figure 4B,D), showing a substantial reduction in wound closure for both CMT-U27 and CF41. Mg cells after 12 h of culture with MitoQ, particularly at concentrations ranging from 5 to 10 µM (Figure 4). Furthermore, the inhibitory effect on cell migration was evident in cells treated with 5–10 µM MitoQ, as evidenced by a distinct decrease in wound closure at 12 h. Although precise delineation of cell movement boundaries in all experimental groups after 24 h of culture with MitoQ proved challenging, it was apparent that cell migration was hindered in cells treated with 5–10 µM MitoQ (Figure 4A,C).

### 2.5. Effects of MitoQ on Canine Mammary Cancer Cell Death Involving Extracellular Signal-Regulated Kinase and Protein Kinase B Signaling

**Figure 5:**
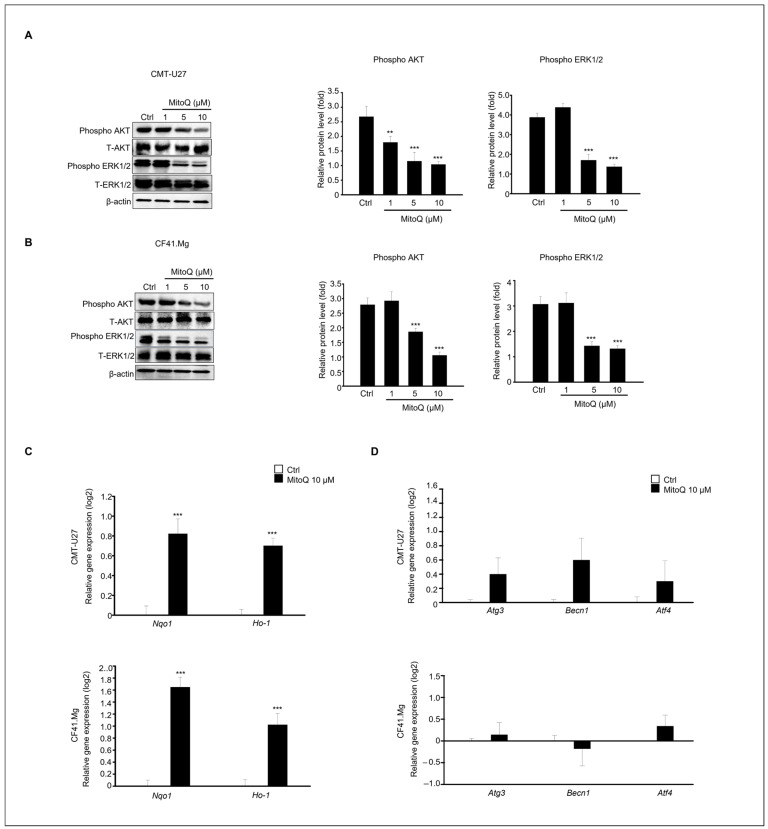
MitoQ suppresses phosphorylation of AKT and ERK1/2 and increases gene expression levels of *Nqo1* and *Ho-1* in both CMT-U27 cells and CF41. Mg cells. Immunoblotting of phosphorylated AKT, Total-AKT, phosphorylated ERK1/2, Total ERK1/2, and β-actin from (**A**) CMT-U27 and (**B**) CF41. Mg cells after MitoQ treatment. The graph represents the quantified protein levels normalized to the total protein form. β-actin is shown as a loading control. The graph presents the relative protein expression levels as mean ± SEM. n = 4, ** *p* < 0.01; *** *p* < 0.001. (**C**) Gene expression levels of *Nqo1* and *Ho-1* in CMT-U27 and CF41. Mg cells. (**D**) Gene expression levels of Atg3, Becn1, and Atf4 in CMT-U27 and CF41. Mg cells. Data show mean ± SD. ** *p* < 0.01; *** *p* < 0.001 compared to the control.

This study investigated the regulation of the AKT and ERK signaling pathways in CMT-U27 and CF41.Mg cell death induced by MitoQ, as these pathways play crucial roles in cell survival, proliferation, and apoptosis. After 24 h of MitoQ treatment in both cell lines, phosphorylation of AKT and ERK1/2 was significantly decreased in CMT-U27 and CF41.Mg cells (Figure 5A,B). Notably, these expression patterns were distinctly observed in the 5–10 μM treatment groups from both cell lines. Additionally, we examined the expression patterns of the antioxidant-related genes, *Nqo1* and *Ho-1*, using qPCR (Figure 5C). The expression levels of both genes were significantly higher in MitoQ-treated samples than in the controls in both cell types. Furthermore, since AKT inhibition promotes autophagy during cancer cell death, we investigated whether the genes associated with autophagy, including *Atg3*, *Becn1*, and *Atf4*, were upregulated or downregulated using qPCR (Figure 5D). The results showed that the expression levels of these genes were not statistically significant.

## 3. Discussion

Reactive oxygen species (ROSs), highly reactive molecules, have garnered attention in diverse cancer therapies. ROSs are natural byproducts of numerous cellular processes [35]. Typically, cancer cells exhibit elevated basal ROS levels compared to healthy cells due to an imbalance between oxidants and antioxidants. ROSs play a dual role in cell metabolism. At low to moderate levels, they act as signal transducers, stimulating cell proliferation, migration, invasion, and angiogenesis [51,52]. However, high ROS levels can inflict damage on proteins, nucleic acids, lipids, membranes, and organelles, ultimately leading to cell death. Extensive research has highlighted promising results of anticancer therapies that modulate ROS levels, such as immunotherapy, both in vitro and in vivo [53]. Mitochondria play crucial roles in regulating metabolic redox alterations within cancer cells, and MitoQ operates differently in cancer cells compared to healthy cells [52].

Our findings unequivocally demonstrate a significant reduction in both CMT-U27 and CF41.Mg cell numbers in MitoQ-containing medium after 24 h of culture, primarily via the apoptotic cell death pathway. Recent evidence supports the anticancer effects of MitoQ. Cheng et al. reported the effective suppression of human breast cancer (MDA-MB-231) and glioma (U87MG) cell proliferation by MitoQ through antioxidation mechanisms [35]. Moreover, both in vitro and in vivo studies have revealed MitoQ’s inhibition of pancreatic and breast cancer metastasis by regulating mitochondrial superoxide. Notably, MitoQ administration successfully prevented the recurrence of human breast cancer in mice xenografted with this cell line [53,54].

In studies involving pancreatic cancer cells, MitoQ concentrations ranging from 100 to 500 nM were deemed clinically relevant. However, for our experiments, we selected a MitoQ concentration range of 1–10 µM, slightly higher than the dosage used in the aforementioned study [54]. This suggests that the effective concentration of MitoQ may vary depending on cell type and species. For instance, Ashutosh Rao et al. reported greater susceptibility of breast cancer cell lines to MitoQ compared to normal mammary cells, as evidenced by lower GI50 values. Specifically, MDA-MB-231, MCF-7, and MCF12A cell lines exhibited GI50 values of 296 nM, 113 nM, and >10 μM for MitoQ, respectively [55].

Oxidative stress (OS) involves highly reactive compounds like ROSs and RNSs, byproducts of oxygen and nitrogen metabolism. These include free radicals such as superoxide (O_2_^−^) and hydroxyl (OH) radicals, as well as non-radicals like hydrogen peroxide (H_2_O_2_) and peroxynitrite (ONOO). They damage cellular components like proteins, lipids, DNA, and carbohydrates, contributing to various diseases [56,57].

In cancer, Ashutosh Rao et al. reported that MitoQ, an antioxidant, induced oxidative stress by increasing oxidation markers and activating the Keap1-Nrf2 pathway. This resulted in heightened Nrf2 activity, leading to autophagy and cell cycle arrest [55].

Both antioxidant and ROS induction have been explored in cancer treatment, albeit through different mechanisms. Cancer cells often harbor higher ROS levels than healthy cells. Inducing additional ROSs can surpass cancer cells’ capacity to manage oxidative stress, inducing cell damage and death. This selective toxicity towards cancer cells has been exploited in various cancer treatment modalities [58,59].

In contrast, antioxidants like MitoQ are believed to safeguard healthy cells from damage caused by excessive ROS, potentially reducing the risk of cancer initiation by neutralizing harmful free radicals. Both strategies aim to manipulate the redox balance within cells, either to shield healthy cells from harm or to selectively target cancer cells. Moreover, the impact of antioxidants varies depending on cancer type, specific treatment, and disease stage [55,60]. Utilizing antioxidants and inducing ROSs in cancer treatment is intricate and context dependent, with both strategies aiming to manipulate cellular redox balance to either protect healthy cells or selectively target cancerous ones. The effectiveness and appropriateness of these approaches hinge on the specific conditions and characteristics of the cancer under treatment.

Our results demonstrated a significant increase in the antioxidant-related genes *Nqo1* and *Ho-1* in MitoQ-treated cancer cells, indicating that MitoQ induces cancer cell death by reducing ROS levels within cancer cells. This likely involves a different mechanism than that in healthy cells. While ROSs induce cell damage in healthy cells, as mentioned earlier, antioxidants like MitoQ enhance cell survival. However, as evidenced by our findings, this pattern did not hold true for CMT-U27 and CF41.Mg cancer cells.

Additionally, the phosphorylation levels of AKT and ERK1/2 markedly decreased in CMT-U27 and CF41.Mg cells after MitoQ treatment. Similarly, Nazarewicz et al. have reported that high mitochondrial ROS levels in cancer cells may contribute to a highly proliferative phenotype [18,19,20]. Lowering ROS levels in cancer cells may dampen the activity of redox-sensitive Akt and ERK [61]. The ERK1/2 and AKT cascades play pivotal roles in triggering various cellular processes, including cell survival and death. It has been noted that the ERK1/2 pathway can regulate BCL-2 protein activity to promote cell survival, and inhibiting ERK1/2 signaling, either directly or indirectly, can induce cancer cell death [62]. Moreover, AKT overexpression has been linked to the development and metastasis of several cancers [63,64], and exposure to AKT inhibitors results in cancer-related death in cervical [65], prostate [66], and pancreatic cancer [67]. Numerous studies have shown that AKT inhibition promotes autophagy in cancer cells [68]; however, MitoQ did not regulate the autophagy pathway in CMT-U27 and CF41. Mg cells in our study [69].

Building upon the favorable outcomes observed in human breast cancer treatment using antioxidants, our research extends these findings to canine mammary gland cancer cells. As mentioned earlier, MitoQ inhibits tumorigenesis and metastasis of human breast cancer cells by effectively suppressing the proliferation of human triple-negative cancer and glioma cells via an antioxidant mechanism [35]. These results suggest that MitoQ may exert anticancer effects in canine models, mirroring observations in human breast cancer and glioma cells. MitoQ has been shown to significantly inhibit both complex I-induced oxygen consumption and proliferation of glioma cells. This inhibition of oxidative phosphorylation (OXPHOS) by MitoQ primarily underlies its antiproliferative effects in cancer cells [35,70]. Consequently, OXPHOS emerges as a crucial molecular target in cancer therapy. Studies have revealed that enhanced OXPHOS can reprogram mitochondrial energy, promoting metastasis in triple-negative breast cancer [71,72].

Beyond cancer, the beneficial effects of MitoQ on various diseases have been previously documented, including the inhibition of prostatic hyperplasia via androgen receptor and NOD-like receptor family pyrin domain-containing 3 inhibition [68,69,70,71,72,73,74]. Additionally, MitoQ has successfully completed phase I safety and phase II clinical trials for diverse diseases, including Parkinson’s disease and hepatitis C. Numerous studies have demonstrated that MitoQ provides protection against oxidative stress-induced conditions in both cell lines and animal disease models [39]. Here, we present, for the first time, the inhibitory and apoptotic effects of MitoQ on canine mammary gland cancer growth. Our findings suggest that MitoQ holds promise as a therapeutic agent in treating canine tumors.

In conclusion, our study delved into the potential therapeutic efficacy of MitoQ, a mitochondria-targeted antioxidant, in canine mammary tumors (CMT). We observed that MitoQ treatment effectively induced cancer cell death, inhibited cell migration, arrested cell cycle progression, and modulated the expression of phosphorylated AKT, ERK, and ROS-related genes in canine mammary tumor cells, specifically CMT-U27 and CF41.Mg cells. These findings underscore MitoQ’s potential as a safe and effective treatment for CMT, offering a novel approach to combating this prevalent canine cancer. Further research is warranted to explore the clinical applicability of MitoQ in CMT treatment and elucidate the underlying mechanisms of its anticancer effects. Additionally, a more comprehensive investigation into the molecular mechanisms of cancer cell death is imperative.

## 4. Materials and Methods

### 4.1. Cell Culture and Treatment

CMT-U27 and CF41.Mg, epithelial-like rounded cells derived from canine mammary gland carcinoma, were procured from ATCC (Manassas, VA, USA). These cells were cultured in Roswell Park Memorial Institute 1640 medium for CMT-U27 cells or Dulbecco’s Modified Eagle’s Medium for CF41.Mg cells, supplemented with 10% fetal bovine serum and 1% penicillin–streptomycin, in a humidified atmosphere containing 5% CO_2_ at 37 °C. MitoQ was obtained from Selleck Chemicals LLC (Houston, TX, USA) and diluted in dimethyl sulfoxide (Sigma Aldrich, Saint Louis, MO, USA) to prepare a 1 M stock solution. This stock solution was further diluted to the desired concentration using cell culture medium prior to cell treatment.

### 4.2. Cell Viability Assay

Cancer cell viability was assessed using a 3-(4,5-dimethylthiazole-2-yl)-2,5-diphenyl tetrazolium bromide (MTT) assay with the EZ-Cytox Viability Assay Kit (Daeil Lab Services Co., Seoul, Republic of Korea, #EZ1000) following the manufacturer’s instructions. The protocol described in previous studies [74,75] was followed. Briefly, both CMT-U27 and CF41.Mg cells were seeded in 96-well plates at a density of 3 × 10^3^ cells/well in culture medium and incubated for 12 h. MitoQ (0–20 µM) was added to the cultured medium and incubated for 24 h. The reagent was then added to the plate as per the manufacturer’s protocol and our previous study [76]. Absorbance was measured at 490 nm using an Epoch spectrophotometer (BioTek, Winooski, VT, USA). The half-maximal inhibitory concentration (IC_50_) of MitoQ was calculated for each canine cancer cell line: CMT-U27 (IC_50_ = 5.36 µM) and CF41.Mg (IC_50_ = 7.25 µM).

### 4.3. Immunostaining

CMT-U27 and CF41.Mg cells were seeded on 18 mm glass coverslips (BD Biosciences, Franklin Lakes, NJ, USA) and cultured with MitoQ concentrations ranging from 0 to 10 µM for 24 h. Subsequently, cells were fixed with 4% paraformaldehyde at 16 °C for 10 min. After fixation, cells were permeabilized in 0.1% Triton X-100 (Sigma Aldrich, Saint Louis, MO, USA) in PBS solution for 10 min at 16 °C. Following permeabilization, samples were incubated with the primary antibody Ki67 (Abcam, Cambridge, UK), which is expressed in the cell nucleus during active phases of the cell cycle, at 4 °C overnight, followed by incubation with the secondary antibody, Alexa Fluor 594 (Thermo Fisher, Waltham, MA, USA), at room temperature for 1 h. The proliferation index was defined as the ratio of the number of Ki67-positive cells to the total number of cells in each field. Cells were analyzed using a fluorescence microscope (Olympus IX73, Tokyo, Japan).

### 4.4. Flow Cytometry

Flow cytometry was employed to measure cell death, cell cycle progression, and assess mitochondrial dysfunction. Annexin V-FITC staining was conducted using a Dead Cell Apoptosis Kit (Thermo Fisher Scientific Inc., Waltham, MA, USA) as previously described [77]. Each cell line was seeded in a six-well plate and cultured with MitoQ for 24 h. The cells were then harvested and stained with Annexin V-FITC and propidium iodide (PI) in the dark. Annexin V-positive cells were detected using flow cytometry (CytoFLEX; Beckman Coulter, Inc., Miami, FL, USA). For the assessment of mitochondrial dysfunction, JC-1 staining (Biotium Inc, Fremont, CA, USA) was performed to measure mitochondrial membrane potential (∆*Ψm*) of CMT-U27 and CF41.Mg cells. Cells were cultured with MitoQ for 24 h, resuspended in 500 μL of 1X JC-1 reagent working solution, and incubated at 37 °C for 15 min, before staining as previously described [76]. Cells were then analyzed using flow cytometry (CytoFLEX, Beckman Coulter, Inc., CA, USA), and cell images were captured using a microscope (Nikon E−800; Nikon, Tokyo, Japan). For the analysis of cell cycle progression, cells were labeled with PI only and analyzed using flow cytometry.

### 4.5. Wound-Healing Assay

CMT-U27 and CF41.Mg cells were seeded in a 24-well plate. Once confluent, cells were manually scratched using a Scar^TM^ Scratcher (SPL, Pocheon-si, Republic of Korea) and rinsed with Dulbecco’s Phosphate Buffered Saline (DPBS, Wellgene, Gyeongsan, Republic of Korea). Subsequently, fresh medium containing MitoQ (0–10 µM) was added. After treatment, cell images were captured using a microscope (Nikon E−800; Nikon, Tokyo, Japan) over 24 h.

### 4.6. Quantitative Polymerase Chain Reaction (qPCR)

Total RNA extraction from CMT-U27 and CF41.Mg cells was performed using the RNeasy Mini Kit (Qiagen, Hilden, Germany) with DNase treatment [78]. Subsequently, cDNA was synthesized using MMLV reverse transcriptase (Thermo Fisher Scientific, Waltham, MA, USA), with the cDNA serving as a template for qPCR. The qPCR procedure and data analysis methods were consistent with our previous study [74]. The specific qPCR primer sequences are provided in Table 1.

### 4.7. Western Blotting

CMT-U27 and CF41.Mg cells were collected after 24 h of culture with MitoQ treatment and lysed using RIPA lysis buffer (Thermo Fisher Scientific, Waltham, MA, USA) supplemented with protease inhibitor mixture (Roche, Rotkreuz, Switzerland). Total protein quantification was performed using the BCA assay kit (Thermo Fisher Scientific, Waltham, MA, USA). Subsequently, 30 µg of total protein was loaded into the wells of 4–16% gradient SDS-PAGE gels (Bio-Rad, Hercules, CA, USA), and proteins were transferred onto PVDF membranes. The membranes were blocked with 5% skim milk and then incubated with primary antibodies in blocking solution (TBS with 0.1% tween-20 + 1% bovine serum albumin) at 4 °C overnight. Following primary antibody incubation, the membranes were incubated with horseradish peroxidase-conjugated secondary antibodies (anti-mouse/rabbit antibodies) for 1 h and then washed with TBS. Band visualization was achieved using Amersham ECL Prime solution (GE Healthcare, Houston, TX, USA) and an iBright Imaging System (Thermo Fisher Scientific, Waltham, MA, USA). β-actin or an inactive form protein served as the normalization control. The antibodies used for immunoblotting are detailed in Table 2.

### 4.8. Statistical Analysis

All data are presented as mean ± standard error of at least three independent experiments conducted in triplicate. Mean differences were assessed using one-way analysis of variance, followed by Tukey’s post hoc test. Statistical analyses were performed using the SPSS statistical package, version 15.0 for Windows (IBM Corp., Somers, NY, USA). Comparisons were considered statistically significant at *p* < 0.05.

## Figures and Tables

**Table 1:** Primers Designed for qPCR Using Canine cDNA.

Gene	Forward Primer	Reverse Primer
*NQO1*	5′-GAAGCCGCAGACCTGGTGAT-3′	5′-GCACTCGCTCGAACCAGCCT-3′
*HMOX1*	5′-CTTTCAGAAGGGCCAGGTGAC-3′	5′-TGCTCGATCTCCTCCTCCAG-3′
*ATG3*	5′-TACCAGACACCACGGCTATG-3′	5′-CCTGCATGGGTGAACTGAAC-3′
*BECN1*	5′-GGCTGAGAGACTGGATCAGG-3′	5′-TGTGCCAGATGTGAAAGGTC-3′
*ATF4*	5′-ACCTTTCTGCAACCACTTCC-3′	5′-TTATGCACTGAGGGATCACG-3′
*GAPDH*	5′-AATTCCACGGCACAGTCAAG-3′	5′-TACTCAGCACCAGCATCACC-3′

**Table 2:** List of primary antibodies used.

Antibody	Manufacturer	Catalog Number	Dilution (Usage)
Cleaved-caspase3	Cell Signaling (Danvers, MA, USA)	#9661	1:2000
Caspase3	Cell Signaling	#9662	1:2000
BAX	Cell Signaling	#5023	1:2000
p-p53	Cell Signaling	#9284	1:2000
p53	Cell Signaling	#2524	1:2000
CDK4	Cell Signaling	#12790	1:2000
CDK2	Cell Signaling	#2546	1:2000
Cyclin D1	Cell Signaling	#2978	1:2000
Cyclin E1	Cell Signaling	#55506	1:2000
P-AKT	Cell Signaling	#4060	1:2000
T-AKT	Cell Signaling	#9272	1:2000
P-erk	Cell Signaling	#9101	1:2000
T-erk	Cell Signaling	#4695	1:2000
Ki-67	Abcam	ab15580	1:200
β-actin	Santa Cruz Biotech (Dallas, TX, USA)	sc47778	1:2000

## Data Availability

The data will be made available upon request.
